# The Genetic Basis of Primary Myelofibrosis and Its Clinical Relevance

**DOI:** 10.3390/ijms21238885

**Published:** 2020-11-24

**Authors:** Elisa Rumi, Chiara Trotti, Daniele Vanni, Ilaria Carola Casetti, Daniela Pietra, Emanuela Sant’Antonio

**Affiliations:** 1 Department of Molecular Medicine, University of Pavia, 27100 Pavia, Italy; chiara.trotti01@universitadipavia.it (C.T.); daniele.vanni01@universitadipavia.it (D.V.); ilaria1985@hotmail.com (I.C.C.); 2 Hematology, Fondazione IRCCS Policlinico San Matteo, 27100 Pavia, Italy; d.pietra@smatteo.pv.it; 3 Hematology, Azienda USL Toscana Nord Ovest, 55100 Lucca, Italy; santantonioemanuela@gmail.com

**Keywords:** myelofibrosis, myeloproliferative, mutation

## Abstract

Among classical *BCR*-*ABL*-negative myeloproliferative neoplasms (MPN), primary myelofibrosis (PMF) is the most aggressive subtype from a clinical standpoint, posing a great challenge to clinicians. Whilst the biological consequences of the three MPN driver gene mutations (JAK2, CALR, and MPL) have been well described, recent data has shed light on the complex and dynamic structure of PMF, that involves competing disease subclones, sequentially acquired genomic events, mostly in genes that are recurrently mutated in several myeloid neoplasms and in clonal hematopoiesis, and biological interactions between clonal hematopoietic stem cells and abnormal bone marrow niches. These observations may contribute to explain the wide heterogeneity in patients’ clinical presentation and prognosis, and support the recent effort to include molecular information in prognostic scoring systems used for therapeutic decision-making, leading to promising clinical translation. In this review, we aim to address the topic of PMF molecular genetics, focusing on four questions: (1) what is the role of mutations on disease pathogenesis? (2) what is their impact on patients’ clinical phenotype? (3) how do we integrate gene mutations in the risk stratification process? (4) how do we take advantage of molecular genetics when it comes to treatment decisions?

## 1. Mutation and Disease Pathogenesis

The so-called “phenotypic driver mutations”, which are those able to drive the myeloproliferative phenotype in PMF, occur in *JAK2*, *CALR*, or *MPL* genes. These mutations were initially described as mutually exclusive, but can indeed coexist in a small proportion of cases [1]. Evidence that mutations in *JAK2*, *CALR*, or *MPL* are sufficient to engender an MPN phenotype has been provided by mouse models, where expression of each mutation alone accurately re-capitulate distinctive features of human disease [2,3,4,5].

In 2005, a single point mutation (V617F) was identified in half of patients with PMF [6,7,8,9]. JAK2 is intimately associated with the cytoplasmic portions of receptors for key hematopoietic cytokines, such as erythropoietin (EPO), thrombopoietin (TPO), and granulocyte colony-stimulating-factor (GCSF). Cytokine receptors are activated by ligand binding, the JAK proteins consequently transphosphorylate one another, which attracts STAT proteins, that are, in turn, phosphorylated by the JAKs. The STATs dimerize and translocate to the nucleus, where they function as a transcription factor to modulate the expression of key genes that regulate proliferation, differentiation, or survival. Normal JAK2 functions to activate intracellular signaling pathways following ligand binding; however, *JAK2* V617F is rendered constitutively active. Indeed, the mutation results in loss of the normal inhibitory function provided by the pseudokinase (JH2) domain upon the active (JH1) kinase domain and causes subsequent cytokine independent cell growth.

In 2006, additional genetic aberrations that perturb JAK/STAT signaling were found in 5–10% of patients with PMF. Mutations in exon 10 of the thrombopoietin receptor *MPL* gene result in amino acid changes of tryptophan 515 to leucine, lysine, or alanine (W515L/K/A), which is located within the cytoplasmic domain, proximal to the transmembrane domain [2,10]. These mutations result in conformation changes of the receptor that mimic the consequences of TPO binding, such that cytoplasmic JAK2 molecules are brought into close proximity, thus promoting their activation, transphosphorylation, and ligand-independent intracellular signaling.

In 2013, mutations in calreticulin (*CALR*), an endoplasmic reticulum (ER) chaperone responsible for the appropriate folding of protein before their trafficking either to the cell surface or for extracellular secretion, were identified in around one-third of patients with PMF [11,12]. *CALR* mutations occur as heterozygous insertions and/or deletions that are all located in the last exon of the gene (exon 9). Although >50 different *CALR* mutations have been identified, all result in a +1 bp frameshift to an alternative reading frame that alters the amino acid composition in the C-terminal part of the protein from acidic residues to basic residues and also leads to a loss of its ER retention signal KDEL. Mutant CALR complexes with MPL and results in its constitutive activation [13,14,15]. The extracellular domain of MPL, along with both the N-terminal and mutant C-terminal of CALR, appear necessary for this interaction, which may be facilitated by the newly acquired positive electrostatic charge within the C terminus [14,16]. *CALR* mutations were originally classified as type 1 (52-bp deletion) and type 2 (5-bp insertion) on the basis that these mutations are the most common, accounting for around 50% and 30% *CALR* mutations, respectively [11]. This classification was later refined to encompass type 1 and type 1-like (65%), type 2 and type 2-like (32%), and other (3%) groups, with these categories defined according to the modification of the alpha-helix structure of the mutant protein, and on the absence or presence of a residual calcium-binding domain, as a consequence of the deletion of negatively charged amino acids stretches in the wild-type *CALR* C terminus [17]. In detail, type 1 and type 1-like mutations result in the deletion of 2 stretches of negatively charged amino acids; type 2 and type 2-like mutations do not result in the deletion of negatively charged residues, and other mutations result in the deletion of 1 stretch of negatively charged amino acids. The classification of *CALR* mutations is relevant for MPN phenotype and prognosis, as will be discussed in the next section.

Patients who do not carry *JAK2* V617F mutation, exon 10 *MPL* mutations, and exon 9 *CALR* mutations are defined as “triple-negative”. A few of these patients carry non-canonical mutations in *JAK2* and *MPL* [18,19]. Therefore, the entire coding region of *JAK2* and *MPL* may need to be covered for a complete diagnostic workup of these selected cases. Overall, 10% of patients with PMF have, as-yet, undiscovered drivers of their disease. Triple-negative MPN is particularly difficult to be diagnosed and differentiated from other myeloid disorders [20]. Recently, nonsense or frameshift mutations in the *MLL3* gene that codes for ad epigenetic modifier (histone methyltransferase) have been reported in a fraction of triple-negative MPN patients [21]. The same kind of mutations have been identified in acute myeloid leukemia (AML) [22], thus suggesting it may act as a tumor suppressor in myeloid neoplasms.

Approximately one-third of patients with PMF harbor additional mutations in known drivers of myeloid malignancies. These mutations impact the process of DNA methylation (*TET2*, *DNMT3A*, *IDH1/2*), chromatin modifications (*ASXL1*, *EZH2*), RNA splicing (*SF3B1*, *SRSF2*, *U2AF1*), and DNA repair (*TP53*).

These observations have been acknowledged from a diagnostic standpoint, so that, in the absence of *JAK2*, *CALR*, or *MPL* mutations, the presence of one of the most frequent accompanying mutations (*ASXL1*, *EZH2*, *TET2*, *IDH1/IDH2*, *SRSF2*, *SF3B1*) is now included among the major criteria for both prefibrotic-myelofibrosis (pre-PMF) and overt-myelofibrosis according to the 2016 World Health Organization (WHO) classification [23], as reported in Table 1. However, some of these mutations (in particular in *TET2*, *DNMT3A*, *ASXL1*) have been found in otherwise healthy persons, especially when older than 65 years, advocating caution in interpreting the results of genotyping for diagnosis in the absence of overt clinical and hematological abnormalities [24]. Then, the identification of these mutations in triple-negative patients should be carefully interpreted, taking into account also the other diagnostic criteria (clinical phenotype and blood cell count, together with bone marrow abnormalities), since they may be signs of an underlying, antecedent clonal hematopoiesis or may be shared with other myeloid neoplasms, as myelodysplastic syndromes (especially those with fibrosis) and low blast count AML [20]. Finally, the mutation profile in triple-negative patients might help in distinguishing true clonal MF from autoimmune MF. Differentiating between a clonal disease and a secondary phenomenon is, indeed, relevant from a therapeutic standpoint, since the latter is sensitive to steroid treatment [25].

## 2. Mutation and Clinical Phenotype

The type of mutation, mutation allele burden, order of acquisition, and combinations of genetic events may have an impact on the disease phenotype.

*JAK2* V617F mutations in PMF are associated with older age, higher HB level, leukocytosis, and lower platelet count [26,27]. Conversely, *CALR* mutations in PMF have been shown to associate with younger age, higher platelet count, less frequent leukocytosis, anemia, and transfusion requirements, fewer spliceosome mutations, and lower dynamic international prognostic scoring system-plus (DIPSS-plus) scores compared with *JAK2*-mutated disease [11,28,29]. Some studies specifically aligned *CALR* type 1 variants with the aforementioned characteristics and phenotypically clustered type 2 variants with *JAK2* mutants [29]. Direct comparison of type 1 and type 2 *CALR* mutations on the basis of hematological and clinical variables has yielded inconsistent findings. *CALR* type 2 mutations have been shown to correlate with higher risk DIPSS-plus scores, marked leukocytosis, and higher circulating blast percentage compared with type 1 variants [29], whereas other studies failed to observe any substantial phenotypic differences [17,30], Triple-negative PMF patients have been shown to be older, with lower Hb levels, platelet and leukocyte counts, and higher IPSS risk [28]. The risk of thrombosis is higher in *JAK2* mutated PMF compared to *CALR* mutated PMF, despite the fact that calreticulin mutated patients display higher platelet counts, both in essential thrombocythemia and in PMF [28,31]. Thus, *JAK2* V617F appears to be thrombophilic also in patients with PMF, as in patients with PV and ET.

Driver mutation allele burden may be another factor that influences the clinical course in PMF. Concerning survival, earlier studies identified a low *JAK2* V617F allele burden as a risk factor for shortened survival in PMF [32,33], as compared to patients whose burden was higher than 50%, suggesting a “burn out phase” of the disease, prone to bone mallow failure”. Otherwise, newer investigations have found older age and high *JAK2* allele burden to be associated with elevated plasma C-reactive protein levels and a pattern of progressive disease, supporting a potential relationship between allelic frequency, inflammation, and disease evolution [34]. Though debated, a high allele burden has also been associated with a greater likelihood of achieving a spleen response under JAK-inhibitor treatment [35]. Concerning the vascular risk, PMF patients with *JAK2* V617F allele burden higher than 75% are high-risk patients as they are prone to develop thrombo-hemorrhagic complications during the disease course. A link between *CALR* mutation allelic burden and disease phenotype has been reported in the literature as it was demonstrated for *JAK2* mutation. A high *CALR* allele burden is more frequent in PMF than in ET [36]. Patients with high *CALR*-mutant burden have acquired copy neutral loss of heterozygosity (LOH) of chromosome 19p, involving the transition from heterozygosity to homozygosity for the *CALR* mutation [11]. Anyway, 19pLOH appears to be a relatively uncommon event [37], in contrast with 9pLOH in *JAK2*-mutant MPN and 1pLOH in *MPL*-mutant MPN [8,38,39]. It has been suggested that the clonal expansion in *CALR*-mutant MPN is faster than that observed in *JAK2*-mutant MPN [40].

Somatic mutations in MPN do not seem to be acquired in a predetermined order as happens in other malignancies, but occur rather randomly [41]. The order of somatic mutation acquisition can be inferred from the genotypes of detectable subclones. For instance, if some tumor cells have *JAK2* V617F, and other from the same patient bear *JAK2* V617F with an additional somatic mutation, then this indicates that *JAK2* V617F came first. The order of acquisition influences clinical presentation as it has been demonstrated for ET and PV: mutations in either *DNMT3A* or *TET2* are associated with an ET phenotype when acquired prior to *JAK2* V617F; by contrast, acquisition of *JAK2* V617F prior to mutation of *DNMT3A* or *TET2* is associated with PV [42,43]. Data regarding the impact of mutation order on clinical phenotype in myelofibrosis are still lacking, though we know that in patients with multiple mutations, *JAK2* V617F was generally an earlier event in those with PMF [21].

Finally, also the germline genetic background might influence the molecular and cellular consequences of nascent PMF clones after their acquisition of phenotypic driver mutations [44].

Targeted sequencing of gene sets in myelofibrosis will be increasingly used in the near future because it will allow profiling of the mutational landscape of each patient and to better define the role of single or multiple, interacting mutations on clinical phenotype and prognosis [21].

## 3. Mutation and Prognosis

Over the past 10 years, prognostic scoring systems have been developed for PMF, including the International Prognostic Scoring System (IPSS) in 2009 [45], the Dynamic International Prognostic Scoring System (DIPSS) in 2010 [46] and the DIPSS-plus in 2011 [47]. They are essentially based on clinical and hematologic parameters, the only genetic parameter being chromosomal abnormalities in the DIPSS-plus.

IPSS utilizes five independent predictors of inferior survival evaluated at initial diagnosis: age > 65 years, hemoglobin <10 g/dL, WBC count >25 × 109/L, circulating blasts ≥1%, and presence of constitutional symptoms. Scores ranging from 0 to ≥3 define four risk groups (low, intermediate 1, intermediate 2, and high-risk) with a corresponding median survival of 11.3, 7.9, 4, and 2.3 years [45].

IPSS was subsequently modified into the DIPSS, which uses the same prognostic variables but assigns 2 points instead of 1 to hemoglobin <10 g/dL and can be applied at any time during the disease course [46].

DIPSS was further refined by incorporating three additional DIPSS-independent risk factors: unfavorable karyotype, PLT count lower than 100 × 10^9^/L, and transfusion requirement. The new prognostic model, DIPSS-plus, identifies four risk groups with median survival ranging from 15.4 to 1.3 years [47].

With the increasing knowledge of the molecular basis of PMF, it is becoming evident that both driver and non-driver mutations have relevance in terms of prognostic significance. PMF carrying *CALR* mutations [28], especially *CALR* type-1 mutations [29], have the best prognosis, whereas “triple-negative” PMF have the worst prognosis [28,48]. However, different mutations may interact with each other and modulate prognosis, as it has been shown for *CALR* and *ASXL1*: the best survival has been, indeed, reported for those patients harboring a *CALR* mutation with wild type *ASXL1*, while patients with the opposite genotype (*CALR* wild type and *ASXL1* mutated) displayed a significantly worse prognosis. A third risk category could be identified, including those patients with both mutations or wild type for both genes [49].

This is somewhat similar to the AML scenario, where both *NPM1* and *FLT3* mutational status do influence prognosis [50].

Mutations in *ASXL1*, *EZH2*, *SRSF2*, or IDH1/2, called high molecular risk (HMR) mutations, have been shown to represent unfavorable prognostic factors, as these genetic lesions identify PMF patients who are at risk for premature death or leukemic transformation independently of the conventional prognostic scoring systems [27]. Moreover, the number of detrimental mutations represents an additional unfavorable prognostic factor, with two or more mutations being associated with shortened leukemia-free survival [51]. The frequency of HMR mutations and their prognostic consequences seems to be somewhat different between the two PMF subtypes: indeed, an HMR status has been less frequently reported in pre-PMF (27.0%) than in overt PMF (44.4%), while the negative prognostic effect of harboring more than one HMR mutations seems to be higher in pre-PMF, though statistically significant in both entities [52]. The presence of HMR mutations is also associated with a shorter time to treatment failure in patients treated with JAK inhibitors [53].

Together with the above-mentioned HMR mutations, genetic events occurring in the RAS/MAPK pathway genes have been recently associated with high-risk clinical features and worse prognosis, compared with wild type patients [54].

Then in 2018, a clinical/molecular prognostic model that includes both driver mutations and detrimental co-operating mutations was created. The Mutation-Enhanced International Prognostic Score System (MIPSS-70) includes the following risk factors: hemoglobin <10 g/dL, abnormal leukocyte count (either <4 or >25 × 10^9^/L), platelet count <100 × 10^9^/L, circulating blasts ≥2%, constitutional symptoms, bone marrow fibrosis grade, IPSS/DIPPS-plus category, driver mutations, absence of *CALR* type 1-like mutation, individual HMR mutations, HMR category, and the presence of ≥2 HMR mutations. Each parameter has a weight of 1 point, except for leukocytosis, thrombocytopenia, and the presence of ≥2 HMR mutations, which are each weighted 2 points. Patients are stratified into three categories: low-risk with a median survival of 27.7 years, intermediate-risk with a median survival of 7.1 years, and high-risk with a median survival of 2.3 years. In the MIPPS-70 plus that includes cytogenetic information, four risk categories are delineated with median survival ranging from 1.7 to 20 years [55]. The primary aim of this new score is to better select patient candidates for allogeneic bone marrow transplantation.

Later two new prognostic systems for PMF were introduced: the MIPSS70+ version 2.0 (mutation- and karyotype-enhanced international prognostic scoring system) and the GIPSS (Genetically Inspired Prognostic Scoring System).

The MIPSS70+ version 2.0 incorporates the recently revised three-tiered cytogenetic risk levels [56], *U2AF1*Q157 as an additional HMR mutation, and new sex- and severity-adjusted hemoglobin thresholds (severe anemia for hemoglobin levels <8 g/dL in women and <9 g/dL in men, moderate anemia for hemoglobin levels of 8–9.9 g/dL in women and 9–10.9 g/dL in men). MIPPS70+ version 2.0 includes five genetic and four clinical variables: very high risk (VHR) karyotype (4 points), unfavorable karyotype (3 points), presence of one (2 points) or ≥2 HMR mutations (3 points), absence of type 1/like *CALR* mutation (2 points), constitutional symptoms (2 points), severe anemia (2 points), moderate anemia, (1 point) and circulating blasts ≥2% (1 point). MIPSS70+ version 2.0 features five risk categories: very high risk (≥9 points); high risk (5–8 points); intermediate risk (3–4 points); low risk (1–2 points); and very low risk (zero points), with corresponding median survivals of 1.8 years, 4.1 years, 7.7 years, 16.4 years and “not reached” [57,58].

**Figure 1:**
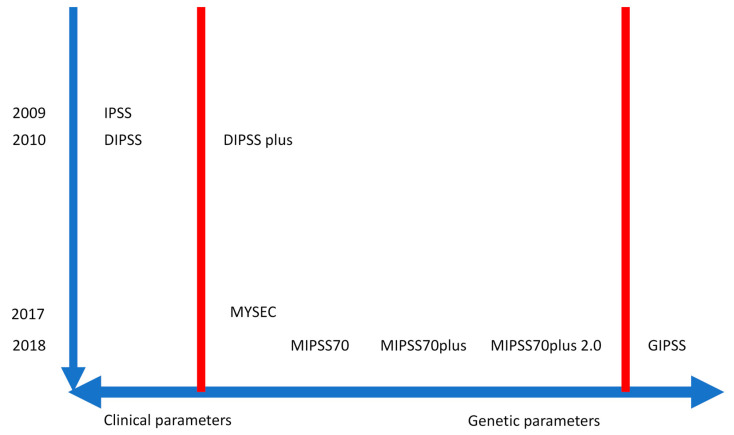
The different scores developed during the last two decades are based only on clinical parameters (IPSS and DIPSS), on clinical and genetic parameters (DIPSS plus, MYSEC, MIPSS70, MIPSS70plus, MIPSS70plus 2.0), or on genetic parameters only (GIPSS).

GIPSS, which is based exclusively on mutations and karyotype, represented the first attempt to model PMF survival on genetic features that fully replace traditional clinical variables, ads VHR karyotype (2 points), unfavorable karyotype (1 point), absence of type 1/like *CALR* mutation (1 point) and presence of *ASXL1* (1 point) *SRSF2* (1 point) and *U2AF1*Q157 (1 point) mutations. The GIPSS features four risk categories: high risk (≥3 points), intermediate-2 risk (2 points), intermediate-1 (1 point), and low risk (zero points), with corresponding median survivals of 2 years, 4.2 years, 8.0 years, and 26.4 years [59]. The currently available prognostic scores are reported in Table 2 and represented in Figure 1.

## 4. Mutation and Treatment Response

### 4.1. Impact on Response to JAK Inhibitors or Interferon

The clinical efficacy of ruxolitinib in myelofibrosis seems not to be affected by the underlying driver mutations [60,61], but may be influenced by non-driver genetic events, especially when present in a high number. Patients with ≥3 mutations from a customized, myeloid neoplasms-associated gene panel have a shorter time to treatment discontinuation and shorter overall survival than those with fewer mutations [62]. Moreover, drug efficacy, in terms of both symptoms and spleen response at 6 months, has been recently reported to be lower in patients harboring mutations in the RAS/MAPK pathway, as *NRAS*, *KRAS*, or *CBL* [54].

Furthermore, drug failure has been associated with signs of clonal evolution in a not negligible percentage of cases, ranging from 17% in a cohort of real-life treated patients to 33% in a clinical trial setting, with a consistently negative impact on patients’ survival [63,64].

In addition, the response to other JAK-inhibitors, such as momelotinib or imetelestat, may be influenced by the presence of *ASXL1/SRSF2* or *ASXL1* mutations, respectively [65,66]. The clinical efficacy of interferon (IFN) in myelofibrosis seems not to be affected by the underlying driver mutations but may be influenced by HMR mutations [67,68].

### 4.2. Impact on Transplant Outcome

Different mutations may have an impact on the outcome after hematopoietic bone marrow transplantation (HSCT). Early smaller reports suggested a benefit for *JAK2* positive patients [69], whereas later studies demonstrated a positive impact for *CALR* positive patients and a negative impact on patients’ prognosis of triple-negative status [70]. However, in those studies, only driver mutations were investigated. To determine the impact of molecular genetics on outcome after HSCT, the group of Kroger screened 169 patients affected with myelofibrosis for 16 frequently mutated genes [71]. *CALR* mutation was an independent factor for lower non-relapse mortality, improved progression-free survival (PFS), and overall survival. *ASXL1* and *IDH2* mutations were independent risk factors for lower PFS, whereas no impact was observed for triple-negative patients. Authors concluded that molecular genetics, especially *CALR*, *IDH2*, and *ASXL1* mutations, may thus be useful to predict outcomes independently from known clinical risk factors after HSCT. Very recently, driver and subclonal mutations were incorporated in the myelofibrosis transplant score (MTSS), a scoring system that includes clinical-molecular and transplant-specific factors, with the aim of predicting posttransplant outcome. Multivariable analysis on survival identified age ≥57 years, Karnofsky performance status <90%, platelet count <150 × 109/L, and leukocyte count > 25 × 109/L prior to transplantation, HLA-mismatched unrelated donor, *ASXL1* mutation and non-*CALR*/*MPL* driver mutation genotype being independent predictors of outcome [72].

### 4.3. Minimal Residual Disease (MRD)

The presence of myelofibrosis-specific molecular markers offers the possibility to monitor the depth of remission after HSCT and to start a preemptive therapy at the first, minimal reappearance of disease [73,74,75]. The importance of the clearance of *JAK2*/*CALR*/*MPL* mutation was recently demonstrated in a cohort of 136 patients who received alloHSCT. Patients with detectable mutations at day +100 or at day +180 had a significantly higher risk of clinical relapse at 5 years than molecular-negative patients (62% vs. 10%, *p* < 0.001) [76]. Minimal residual disease (MRD) detection is clinically relevant as it may prompt a reduction of immunosuppressive agents or a donor-derived leukocyte infusion (DLI). The latter, in detail, is more effective when used for a molecular rather than for a clinical relapse [77]. The sensitivity of the method to detect MRD is pivotal. For *JAK2* and *MPL*, a sensitivity of 10^−5^ has been described by PCR technique [73,74].

Notably, a JAK2 V617F qPCR assay was generally recommended because of its sensitivity and consistent performance across different qPCR platforms [78,79,80].

The use of *CALR* mutation as a marker of MRD is more problematic due to the variety of indels reported in the *CALR* gene and the scarce quantification accuracy of fragment length analysis and Sanger sequencing. Both a sensitive qPCR approach and a digital PCR assay that allows the accurate determination of *CALR* allelic burden for the most frequent alterations (type 1 and type 2) have been developed [81,82]. In addition, very sensitive digital PCR assays have been described for the detection of low *JAK2* and *CALR* mutant burdens [83].

Subclonal mutations, if present, could be extremely useful to monitor MRD, both in triple-negative patients or as an additional tool for those who have a traditional driver event. In this regard, next-generation sequencing (NGS) approaches for MRD monitoring might be hopefully developed in the future.

## 5. Conclusions

The rapidly expanding field of molecular genetics for myelofibrosis has opened new horizons in the diagnosis, prognosis, treatment decision-making, and monitoring of this disorder. Recommendations for molecular testing in MPN has been recently provided [84] and include both driver and non-driver genetic events. The latter may aid in the diagnostic process of patients who tested negative for the three driver mutations, in which an extended mutation panel including at least *ASXL1*, *EZH2*, *IDH1*/2, and *SRSF2* is recommended. The same extended panel is also appropriate for prognostic prediction, in particular for patients classified as intermediate risk, for which treatment allocation, counseling and/or frequency of monitoring may be refined according to the individual genetic background. High-sensitivity quantitative monitoring of *JAK2* V617F and *CALR* mutations, or quantitative evaluation of another clonal marker in triple-negative patients, are recommended in all patients after HSCT for MRD assessment.

While comprehensive gene sequencing of hematological cancer patients is becoming an increasingly accessible event for routine clinical use, many uncertainties still remain. As an example, how many times should PMF patients be tested? In fact, MPNs are dynamic diseases, so that high-risk genomic features may be gained over time, requiring treatment intervention (or HSCT consideration).

Genome profiling will likely become the basis for a personally-tailored prognosis prediction, but we acknowledge that clinical translation of recent scientific advances is still a work in progress.

## Figures and Tables

**Table 1:** The revised 2016 World Health Organization (WHO) diagnostic criteria for primary myelofibrosis.

Overt Primary Myelofibrosis (All 3 Major and at Least 1 Minor are Required)	Prefibrotic Primary Myelofibrosis (All 3 Major and at Least 1 Minor are Required)
Major criteria Presence of megakaryocytic proliferation and atypia, accompanied by either reticulin and/or collagen fibrosis grades 2 or 3 Not meeting WHO criteria for *BCR-ABL1-*positive chronic myeloid leukemia, polycythemia vera, essential thrombocythemia, myelodysplastic syndromes, or other myeloid neoplasms Presence of *JAK2*, *CALR* or *MPL* mutation or in the absence of these mutations, presence of another clonal marker,^†^ or absence of minor reactive BM reticulin fibrosis	Major criteria Megakaryocytic proliferation and atypia, without reticulin fibrosis >grade 1, accompanied by increased age-adjusted bone marrow cellularity, granulocytic proliferation, and often decreased erythropoiesis Not meeting WHO criteria for BCR-ABL1-positive chronic myeloid leukemia, polycythemia vera, essential thrombocythemia, myelodysplastic syndromes, or other myeloid neoplasms Presence of *JAK2*, *CALR* or *MPL* mutation or in the absence of these mutations, presence of another clonal marker,^†^ or absence of minor reactive BM reticulin fibrosis
Minor criteria Anemia not otherwise explained Leukocytosis (WBC count ≥ 11 × 109/L) Palpable splenomegaly Lactate dehydrogenase (LDH) level increased to the above-upper-normal limit of the institutional reference range Leukoerythroblastosis	Minor criteria Anemia not otherwise explained Leukocytosis (WBC count ≥ 11 × 109/L) Palpable splenomegaly Lactate dehydrogenase (LDH) level increased to the above-upper-normal limit of the institutional reference range

^†^ In the absence of any of the three major clonal mutations, the search for the most frequent accompanying mutations (e.g., *ASXL1*, *EZH2*, *TET2*, *IDH1/2*, *SRSF2*, *SF3B1*) are of help in determining the clonal nature of the disease.

**Table 2:** Prognostic models in primary myelofibrosis.

Prognostic Model	Risk Groups and Clinical Relevance
**International Prognostic Scoring System (IPSS)** [45] IPSS estimates survival at the time of diagnosis.	
Risk factors (weight): Age >65 years (1 point) Constitutional symptoms (1 point) Hemoglobin <10 g/dL (1 point) WBC count >25 × 10^9^/L (1 point) Circulating blasts ≥1% (1 point)	Low risk: 0 (median survival 11.3 years) Intermediate-1 risk: 1 point (7.9 years) Intermediate-2 risk: 2 points (4.0 years) High risk: ≥3 points (2.3 years)
**Dynamic International Prognostic Scoring System (DIPSS)** [46] DIPSS can be applied anytime during clinical course.	
Risk factors (weight): Age >65 years (1 point) Constitutional symptoms (1 point) Hemoglobin <10 g/dL (2 points) WBC count >25 × 109/L (1 point) Circulating blasts ≥1% (1 point)	Low risk: 0 (median survival: not reached) Intermediate-1 risk: 1 point (14.2 years) Intermediate-2 risk: 2 points (4.0 years) High risk: ≥3 points (1.5 years)
**DIPSS-plus** [47] DIPSS-plus can be applied anytime during the clinical course.	
Risk factors (weight): DIPSS score (DIPPS low = 0, DIPPS int-1 = 1 point, DIPPS int-2 = 2 points, DIPSS high = 3 points) RBC transfusion need (1 point) PLT count <100 × 109/L (1 point) Unfavorable karyotype (1 point)	Low risk: 0 (median survival: 15.4 years) Intermediate-1 risk: 1 point (6.5 years) Intermediate-2 risk: 2 points (2.9 years) High risk: ≥3 points (1.3 years)
**MIPSS70** [55] MIPSS70 is used to better select patients <70 years as candidates for allogeneic stem cell transplantation.	
Risk factors (weight): One HMR mutation (1 point) ≥2 HMR mutation (2 points) Type 1/like CALR absent (1 point) Hb <10 g/L (1 point) WBC > 25 × 109/L (2 points) PLT count <100 × 109/L (2 points) Circulating blasts ≥2% (1 point) Constitutional symptoms (1 point) Bone marrow fibrosis ≥2 (1 point)	Low risk: 0–1 points (median survival: 27.7 years) Intermediate risk: 2–4 points (7.1 years) High risk: ≥5 points (2.3 years)
**MIPSS70+ version 2.0** [57] MIPSS70+ version 2.0 incorporates the revised cytogenetic risk levels, U2AF1Q157 as an additional HMR mutation, and new sex- and severity-adjusted hemoglobin thresholds.	
Risk factors (weight): VHR karyotype (4 points) Unfavorable karyotype (3 points) ≥2 HMR mutation (3 points) One HMR mutation (2 points) Type 1/like CALR absent (2 points) Severe anemia (2 points) Moderate anemia (1 point) Circulating blasts ≥2% (1 point) Constitutional symptoms (2 points)	Very low risk: 0 (median survival: not reached) Low risk: 1–2 points (16.4 years) Intermediate risk: 3–4 points (7.7 years) High risk: 5–8 points (4.1 years) Very high risk: ≥9 points (1.8 years)
**GIPSS** [59] GIPSS may be useful in early-stage patients because it can predict outcomes in the absence of clinical signs of progressive disease.	
Risk factors (weight): VHR karyotype (2 points) Unfavorable karyotype (1 point) Type 1/like CALR absent (1 point) ASXL1 mutation (1 point) SRSF2 mutation (1 point) U2Af1Q157 mutation (1 point)	Low risk: 0 (median survival: 26.4 years) Intermediate-1 risk: 1 point (8 years) Intermediate-2 risk: 2 points (4.2 years) High risk: ≥3 points (2 years)
